# Protective Effects of Melatonin Against Zearalenone Toxicity on Porcine Embryos *in vitro*

**DOI:** 10.3389/fphar.2019.00327

**Published:** 2019-04-05

**Authors:** Yao Xu, Kun-Huan Zhang, Ming-Hong Sun, Mei Lan, Xiang Wan, Yu Zhang, Shao-Chen Sun

**Affiliations:** College of Animal Science and Technology, Nanjing Agricultural University, Nanjing, China

**Keywords:** embryo, melatonin, zearalenone, oxidative stress, DNA damage

## Abstract

Zearalenone (ZEA) is an estrogenic mycotoxin produced by Fusarium fungi commonly found in corn, wheat, and other cereals which can infect food and feed commodities, and ZEA mainly has reproductive toxicity which causes widely reproductive disorders in pigs and other animals. However, the toxicity and the functional ways of ZEA on early embryo development is still unclear. In present study we showed that exposure to ZEA (10 μM) significantly decreased the 2-cell and blastocyst developmental rate in porcine early embryos *in vitro*. ZEA treatment resulted in the occurrence of oxidative stress, showing with increased reactive oxygen species (ROS) level, following with aberrant mitochondrial distribution. Moreover, we found positive signals of γH2A.X in the ZEA-treated embryos, indicating that ZEA induced DNA damage, and the increased autophagy confirmed this. These results suggested that ZEA induced oxidative stress, which further caused mitochondria dysfunction and DNA damage on early embryonic development. We next investigated the effects of melatonin on the ZEA-treated embryo development, and we found that melatonin supplementation could significantly ameliorate ZEA-induced oxidative stress, aberrant mitochondria distribution and DNA damage. In all, our results showed that ZEA was toxic for porcine embryos cultured *in vitro* and melatonin supplementation could protect their development from the effects of ZEA.

## Introduction

The early embryo development quality is one of the prerequisites for the success of embryo implantation, which is the threshold to determine the further development after implantation (Teh et al., 2016). The development of mammalian early embryo includes the stages from zygote to blastocyst, while important morphological changes such as cell proliferation, compaction and blastocyst formation are required. During the 8-16-cell stage, when the morula embryo undergoes compaction (Oestrup et al., 2009); while the blastocoel is formed in 16-32-cell, and the blastomeres differentiates into trophectoderm (TE) and inner cell mass (ICM) (Hyttel and Niemann, 1990). Mitochondria are dynamic organelles which are important determinants of oocyte development, fertilization and preimplantation embryo development in mammals (Nagai et al., 2004). They are the primary energy-generating system, which regulate calcium homeostasis, fatty acid oxidation, signal transduction, cell death pathways, and metabolism of different biomolecules (Romek et al., 2011). Mitochondria are main sites of ROS production, and mitochondrial abnormalities may cause excessive oxidative stress (Chen et al., 2003). Maintaining redox dynamic balance is very important for oocyte and embryo production. Higher ROS may change several redox pathways and may eventually lead to DNA damage and apoptosis of oocytes and embryos (Agarwal et al., 2005).

Zearalenone (ZEA) is an estrogenic mycotoxin produced by Fusarium fungi from corn, wheat and other cereals. Animals and humans are widely exposed to ZEA after eating food products that are contaminated with ZEA. ZEA is a non-steroidal estrogen Fusarium mycotoxin that has strong oestrogenic effects due to its competition with 17-B-estradiol for binding to cytosolic estrogen receptors in the uterus, hypothalamus, mammary gland and pituitary gland (Zhu et al., 2014). ZEA mainly has reproductive toxicity, which can cause reproductive disorders in pigs and cattle such as ovarian atrophy, prolong oestrus cycle, persistent luteal body, false pregnancy and abortion, lower litter size or weak litter size (Tiemann and Danicke, 2007). Previous studies have shown that ZEA induced the apoptosis of granulosa cells (Lai et al., 2015), and ZEA also affected spindle morphology, actin filament expression, epigenetic modifications and cortical granule free domain formation of pig oocytes (Zhu et al., 2014; Han et al., 2015). However, till now the toxicity of ZEA on early embryos remains unclear, and the approaches to alleviate the toxicity caused by ZEA exposure has been poorly understood.

Melatonin (5-methoxy-N-acetyltryptamine) is a hormone and secreted principally by the pineal gland at night under normal light/dark conditions (Chern et al., 2012; Claustrat and Leston, 2015). And it was involved in the regulation of biological rhythms and seasonal reproduction in mammals (Reiter et al., 2016). In addition, many studies have shown that melatonin alleviated oxidative stress, reduced apoptosis, as well as regulating cytoskeletal organization (Benitez-King, 2006; Zhang and Zhang, 2014). Melatonin has important roles in reproduction, for example, it can effectively maintain the health morphology of oocytes, delay the decline of mitochondrial membrane potential of aging oocytes, induce oocyte maturation and ensures oocyte merit and quality and promote embryonic development. Compared with other antioxidants, melatonin has the advantages of fast metabolism and less harm to oocytes (Nikmard et al., 2017; Wang et al., 2017). Previous studies showed that melatonin supplement improved the maturation of oocytes under mono-(2-ethylhexyl) phthalate (MEHP) and deoxynivalenol (DON) exposure through its effects on oxidative stress-mediated apoptosis and autophagy rescue (Lan et al., 2018; Zhang et al., 2018). However, it was unclear whether melatonin has protective effects on the porcine embryonic development with the ZEA exposure.

In present study, we adopted parthenogenetic porcine embryos as a model to explore the toxic effects of ZEA on early embryos, and we also explored whether melatonin could alleviate and protect ZEA-effected embryos. Our results showed that melatonin protects early embryo development from the exposure of ZEA by reducing oxidative stress, mitochondria dysfunction and DNA damage.

## Materials and Methods

### Antibodies and Chemicals

Rabbit polyclonal anti-microtubule-associated protein 1 light chain 3 (LC3) antibody was from Cell Signaling Technology (Devers, MA, United States, #ab52768). Rabbit monoclonal to gamma H2A.X (γH2A.X) was from Abcam (Cambridge, United Kingdom, #ab81299). Alexa Fluor 594 goat anti-rabbit antibody, Alexa Fluor 488 were from Invitrogen (Carlsbad, CA, United States). ZEA (#ab142473) purchased from Abcam. If not specifically marked, all other chemicals and reagents were from Sigma-Aldrich Corp.

### Oocyte Collection and *in vitro* Maturation

All protocols performed were approved by the Animal Care and Use Committee of Nanjing Agriculture University and were performed in accordance with Animal Research Institute Committee guidelines. Porcine ovaries were collected from the local slaughterhouse and then transported to laboratory within 3 h in sterile saline (0.9% NaCl) containing 0.03 g/mL of penicillin and 0.03 g/mL of streptomycin at 37°C. Cumulus-oocyte complexes (COCs) were extracted from 3 to 6 mm follicles of ovaries by aspirating with a 20-gauge needle attached to a 5-ml disposable syringe. Oocytes with compact cumulus cells and a uniform ooplasm were selected for *in vitro* maturation (IVM). The COCs was washed three times with IVM medium [TCM199 (St. Louis, MO, United States,# M2154) supplemented with 75 μg/ml of penicillin, 50 μg/ml of streptomycin, 0.5 μg/ml of LH, 0.5 μg/ml of FSH, 10 ng/ml of epidermal growth factor (mouse EGF, Sigma, #E4127), and 0.57 mM cysteine] and cultured in each well of a four-well dish (Nunc, Roskilde, Denmark) containing 500 μl of IVM medium covered with 200 μl mineral oil at 38.5°C in a humidified atmosphere of 5% CO_2_ incubator for IVM. After 42–46 h cultivation, COCs were transferred to 0.1% hyaluronidase (w/v) for 3 min at 38.5°C. After three to four rinses with TCM199, matured MII oocytes were collected for next treatment.

### Production of Parthenogenetic Activation (PA) Embryos

These collected oocytes were washed three times with 38.5°C activation solution (F+ consist of 0.3 M Mannitol, 0.5 M HEPES, 0.1 M MgSO_4_•7H_2_0, 0.1 M CaCl_2_). The oocytes were then electrically activated in microslide 0.5-mm fusion chamber using a single direct current pulse of 0.9 kV/cm for 120 ms (CRY-3B Cell fusion instrument, Ningbo, China), followed by chemical activation with Cytochalasin B (CB, 5 mg/ml) and Cycloheximide (CHX, 1 mg/ml) in PZM-3 medium for 4 h. The control group, ZEA group and ZEA+MEL group were then cultured in PZM-3 medium in a 4-well plate, both at 38.5°C in a 5% CO_2_ at maximum humidity. Cleavage and blastocyst formation percent were examined on 24 and 144 h after activation. After 24 h of culture, 2-cell embryos were used for follow-up studies.

### ZEA and Melatonin Treatment

The parthenogenetic activated oocytes were divided into three groups; (i) control group (Control); (ii) treatment group with ZEA (ZEA); (iii) treatment group with melatonin during ZEA exposure (ZEA+MEL). Dissolution of powdered ZEA with DMSO into concentration of 50 mM and then was diluted to final concentration of 5 and 10 μM in per well with a final volume of 500 μl of IVM medium. The final concentration of DMSO is less than 1% during IVM. Melatonin was dissolved in anhydrous ethanol to 0.1 M then diluted to final concentration of 0.1 μM.

### Immunofluorescence Staining and Confocal Microscopy

The 2-cell embryos were immobilized with 4% (w/v) paraformaldehyde in PBS 30 min and then permeabilized with 1% Triton X-100 (in PBS) for 8–12 h at room temperature, where after blocked by blocking buffer (1% BSA-addition of PBS) 1 h at room temperature to inhibit the non-specific binding of IgG. For LC3 or γH2A.X staining, embryos were incubated with primary antibodies (LC3, 1:500; γH2A.X, 1:200) overnight at 4°C. After washing three times with PBS, the embryos were incubated at room temperature for 1 h with goat anti-rabbit IgG. The embryos were stained with Hoechst 33342 (10 mg/mL in PBS) for 15 min. Finally, samples were mounted on glass slides, and examined with a confocal laser-scanning microscope (Zeiss LSM 700 META, Germany). Image J software is used to analyze the fluorescence intensity. In order to avoid errors, the embryos of the treatment group and the control group were sealed on a glass sheet and scanned with the same parameters to standardize the different repetition. Image J was used to calculate the average fluorescence intensity per unit area of the target area. When we count the fluorescence intensity, we exclude the abnormal embryos, that is, the embryos with very strong and very weak fluorescence intensity. The average fluorescence intensity of all the embryos was taken as the final average fluorescence intensity.

### Detection of Reactive Oxygen Species (ROS) and Mitochondria

To determine the level of ROS in living embryos DCFH diacetate (DCFHDA) kit (Beyotime, China) was used. Embryos were incubated in PZM-3 medium with DCFHDA (1:800) for 30 min at 38.5°C in 5% CO_2_ incubator. After wash embryos three times and fluorescent signals examined with microscope (CKX53, Olympus, Japan). Embryos were incubated in PZM-3 medium with Mito-Tracker Red CMXRos (1:200) (Cat #M7512, Invitrogen, Eugene, OR, United States) at 38.5°C for 30 min. Then wash the embryo three times with the PZM-3 culture medium and examined with a confocal laser-scanning microscope (Zeiss LSM 700 META, Germany).

### Statistical Analysis

At least three replicates were performed in all experiments and no less than 15 embryos were examined with results expression as means ± SEMs. Statistical analyzes were performed using GraphPad Prism software (version 5.0, GraphPad Prism software Inc., San Diego, CA, United States). Statistical comparisons were made by independent sample *t*-tests. A *p*-value of <0.05 was considered significant. The fluorescence pixel intensities were analyzed using Image J software (version 1.50; National Institutes of Health, Bethesda, MD, United States).

## Results

### Melatonin Protects Porcine Early Embryo Development From ZEA Exposure

**FIGURE 1:**
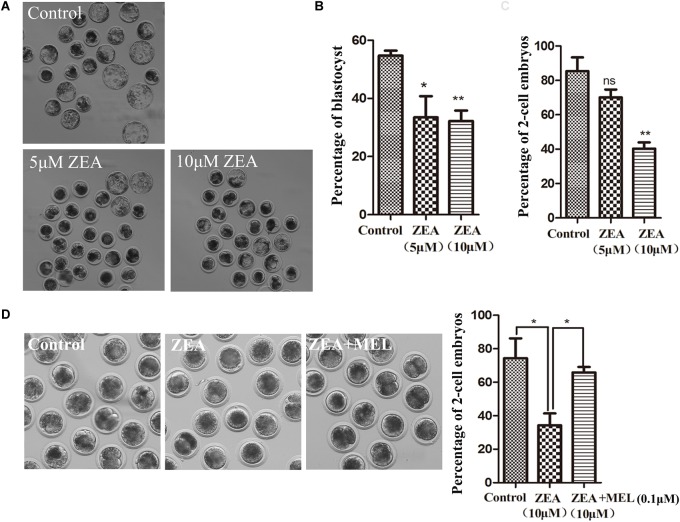
The effect of ZEA on blastocyst and 2-cell formation and protective effect of melatonin. **(A)** Representative images of blastocyst formation in control group and ZEA treatment group with different concentrations. **(B)** Blastocyst percent recorded in control group and ZEA treatment group. Data were presented as mean percentage ± SEM from at least three independent experiments. ^∗^*P* < 0.05 and ^∗∗^*P* < 0.01. **(C)** Percentage of 2-cell in control and different concentrations ZEA treatment groups. **(D)** Representative images of 2-cell formation in control, ZEA-exposed, and ZEA+MEL groups. Percentage of 2-cell in control, ZEA-exposed, ZEA+MEL groups. Data were presented as mean percentage ± SEM from at least three independent experiments. ^∗^*P* < 0.05 and ^∗∗^*P* < 0.01.

Previous studies on porcine oocytes have shown that 10 μ M ZEA significantly affects oocyte maturation (Lu et al., 2018). Therefore after parthenogenetic activation, we first examined the effects of 5 or 10 μM ZEA treatment on embryonic development. After 144 h of culture, the development of blastocysts was observed. As shown in Figure 1A, most embryos were developed to blastocysts in the control group, however, few embryos developed to blastocysts in ZEA treatment groups. Compared with the control group (54.71 ± 11.83%, *n* = 136), the percentage of blastocysts was decreased significantly after ZEA treatment. The proportion of blastocysts was 34.47 ± 1.70% (*n* = 127, *P* < 0.05), 32.22 ± 3.62% (*n* = 122, *P* < 0.01) in 5 and 10 μM, respectively (Figure 1B). These results suggest that ZEA decreased embryo development. We also examined the 2-cell developmental percent after ZEA treatment. As shown in Figure 1C, the 2-cell percent of control group, 5 μM ZEA group and 10 μM ZEA group were 85.34 ± 8.02% (*n* = 136), 70.15 ± 4.52% (*n* = 127, *P* > 0.05) and 40.28 ± 3.64% (*n* = 122, *P* < 0.01), respectively. Next, we explored whether melatonin had the protective effect against ZEA-induced embryonic abnormalities. The embryos were cultured in 10 μM ZEA and 0.1 μM melatonin medium. The results showed that ZEA affected the 2-cell embryo development compared with the control group (74.34 ± 11.83%, *n* = 170 vs. 34.33 ± 7.06%, *n* = 174, *P* < 0.05), while ZEA+MEL group increased the percentage of 2-cell embryos with ZEA group (65.84 ± 3.24%, *n* = 178, *P* < 0.01; Figure 1D).

### Melatonin Prevents Mitochondrial Dysfunction Induced by ZEA in Porcine Embryos

**FIGURE 2:**
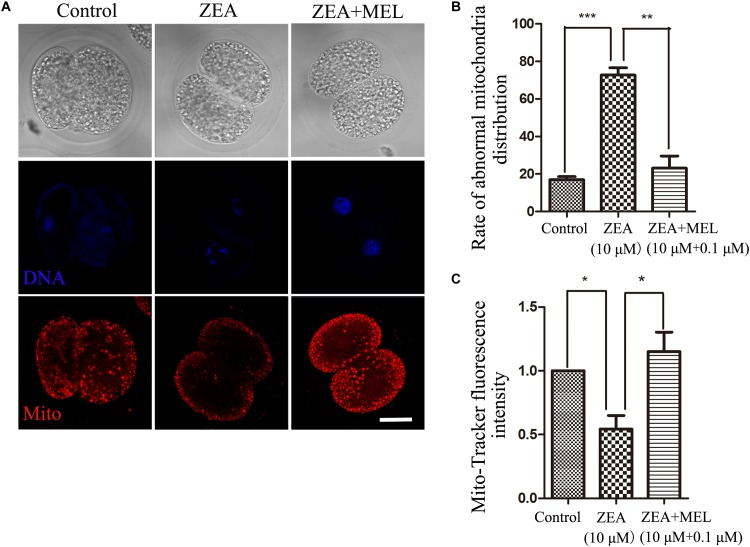
The effect of ZEA and co-exposure of ZEA and melatonin on mitochondria of embryo. **(A)** Representative images of Mito-Tracker (red) fluorescence in the embryos of the control, ZEA-exposed, ZEA+MEL groups. Bar = 20μm. **(B)** Abnormal rate of mitochondria distribution in the control, ZEA-exposed, ZEA+MEL groups. ^∗∗^*P* < 0.01 and ^∗∗∗^*P* < 0.001. **(C)** Mito-Tracker fluorescence intensity in control, ZEA-exposed, ZEA+MEL groups, and Fluorescence images were measured by confocal microscope with the same settings and parameters. ^∗^*P* < 0.05.

To explore the possible causes of ZEA toxicity on embryo development, we examined mitochondrial dysfunction in 2-cell embryos using Mito-Tracker Red CMXRos. As shown in Figure 2A, the mitochondria signals were distributed in the cytoplasm of blastomeres of 2-cell embryos, however, mitochondria distribution showed abnormal pattern in the ZEA treatment group, the signals were attenuated and decreased in the cytoplasm compared with that in the control group. We then examined this in the ZEA+MEL group, and we found that melatonin could protect mitochondria function after ZEA treatment, showing with normal mitochondria distribution. The abnormal rate of mitochondria distribution in the ZEA group was significantly higher than the control group (control group, 16.99 ± 1.66%, *n* = 23; ZEA group, 72.70 ± 3.90%, *n* = 21, *P* < 0.001), while the abnormal rate in the melatonin supplement group significantly lower than the ZEA group (ZEA+MEL group, 23.15 ± 6.48%, *n* = 22, *P* < 0.01) (Figure 2B). Moreover, the relative fluorescence intensity of Mito-Tracker analysis also confirmed this (control group, 1.00, *n* = 19; ZEA group, 0.54 ± 0.11, *n* = 19, *P* < 0.05; ZEA+MEL group, 1.15 ± 0.15, *n* = 18, *P* < 0.05) (Figure 2C).

### Melatonin Alleviates Oxidative Stress Induced by ZEA in Porcine Embryos

**FIGURE 3:**
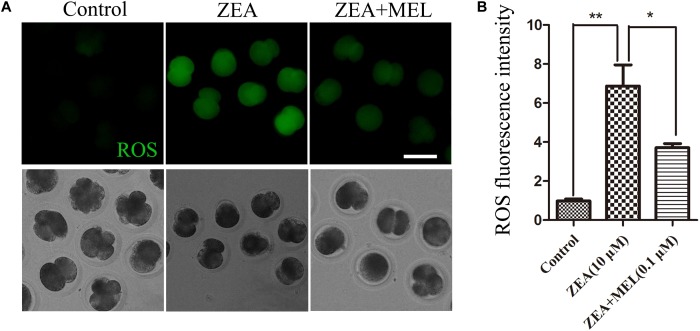
The effect of ZEA and co-exposure of ZEA and melatonin on ROS levels in the porcine embryos. **(A)** Representative images of DCHF-DA fluorescence (green) in the embryos from the control, ZEA-exposed, and ZEA + MEL groups. Bar = 50 μm. **(B)** ROS fluorescence intensity in control, ZEA-exposed, and ZEA+MEL groups, and Fluorescence images were measured by microscope with the same settings and parameters. ^∗^*P* < 0.05 and ^∗∗^*P* < 0.01.

Since mitochondria dysfunction could induced oxidative stress, we then used DCFH-DA fluorescent dye staining to detect ROS levels in the control and ZEA exposed groups in the 2-cell stage. The results of fluorescence staining showed that the level of ROS was significantly higher in the ZEA-exposed embryos than that in the control group. Since melatonin is involved in the redox homeostasis, we also examined the ROS level in the melatonin supplement group, and the results showed that the ROS generation in melatonin-treated group was significantly decreased (Figure 3A). Quantitative analysis of the ROS relative fluorescence intensity also confirmed that present of melatonin significantly reduced ROS levels in 2-cell embryo. As shown in Figure 3B, compared with control group (0.98 ± 0.09, *n* = 22), the ROS relative fluorescence intensity was raised significantly after ZEA treatment (6.88 ± 1.08, *n* = 19, *P* < 0.01), and decreased significantly in ZEA+MEL group (3.71 ± 0.21, *n* = 19, *P* < 0.05).

### Melatonin Alleviates DNA Damage Induced by ZEA in Porcine Embryos

**FIGURE 4:**
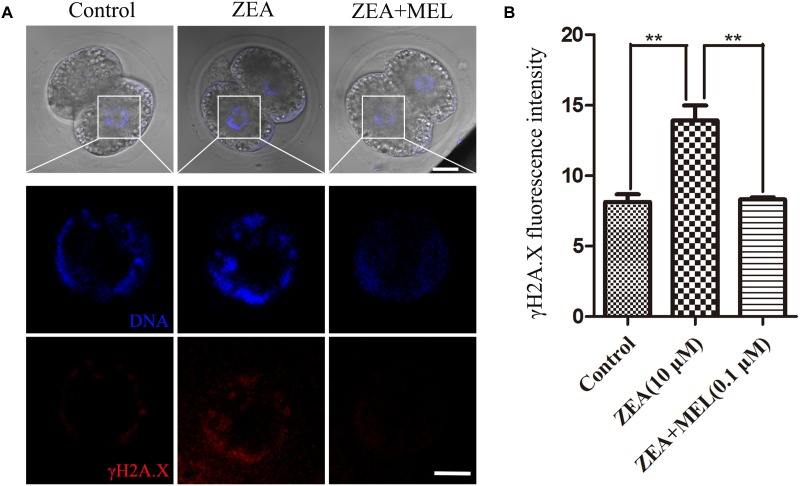
The effect of ZEA and co-exposure of ZEA and melatonin on DNA damage in porcine embryos. **(A)** Representative images of γH2A.X (red) in the embryos from the control, ZEA-exposed, and ZEA+MEL groups. Bar = 20 μm. **(B)** γH2A.X fluorescence intensity in control, ZEA-exposed, and ZEA+MEL groups, and fluorescence images were measured by confocal microscope with the same settings and parameters. ^∗∗^*P* < 0.01.

We next examined whether the embryos had DNA damage, since oxidative stress generally induces DNA damage. As shown in Figure 4A, in the control embryos, there was no signal of γH2A.X in the nucleus, while in the ZEA-treated embryos we found positive signal of γH2A.X. We also examined the ZEA+MEL group, and there was no signal of γH2A.X. The fluorescence intensity of γH2A.X in ZEA group was significantly higher than that in control group whereas fluorescence intensity was markedly decreased in the presence of melatonin (control group, 8.13 ± 0.55, *n* = 22; ZEA group, 13.93 ± 1.06, *n* = 19, *P* < 0.01; ZEA+MEL group, 8.32 ± 0.16, *n* = 19, *P* < 0.01) (Figure 4B).

### Melatonin Reduces Autophagy Induced by ZEA in Porcine Embryos

**FIGURE 5:**
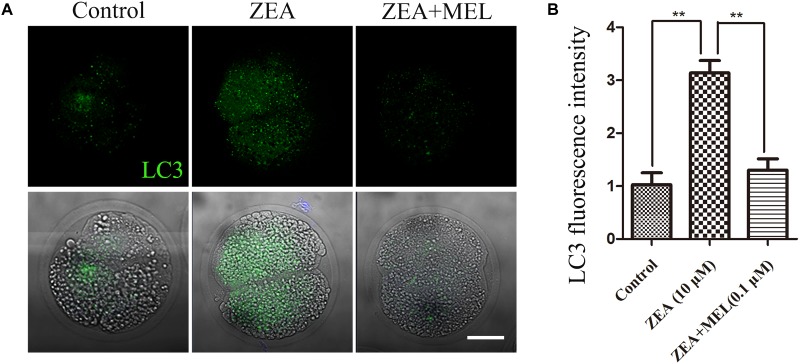
The effect of ZEA and co-exposure of ZEA and melatonin on autophagy in ZEA exposed porcine embryos. **(A)** Representative images of LC3 (green) in the embryos from control, ZEA-exposed, and ZEA+MEL groups. Bar = 20 μm. **(B)** LC3 fluorescence intensity in control, ZEA-exposed, and ZEA+MEL groups, and fluorescence images were measured by confocal microscope with the same settings and parameters. ^∗∗^*P* < 0.01.

Autophagy could eliminate oxidative stress-induced mitochondria. Next, we performed LC3 antibody staining to examine autophagy status. Immunofluorescence staining showed that LC3 increased in the embryos of ZEA group compared with the control group, dots signals were enriched in the cytoplasm of the blastomeres of the 2-cell embryos; while there is barely signals of LC3 in the melatonin supplement group. And the fluorescence intensity of LC3 analysis confirm this (Figure 5A). As shown in Figure 5B, fluorescence intensity analysis of LC3 in ZEA group (3.139 ± 0.23, *n* = 24, *P* < 0.01) was significantly higher than that in control group (1.024 ± 0.22, *n* = 24, *P* < 0.01) and ZEA+MEL group (1.303 ± 0.21, *n* = 21, *P* < 0.01).

## Discussion

In present study we clarified the toxic effects of ZEA on early embryonic development and its possible functional ways, and we also confirmed that melatonin can alleviate and protect the development defects of early embryo induced by ZEA exposure.

The effects of mycotoxins on reproduction are widely known, and several reports showed that some mycotoxins disturb embryo development. T-2 toxin and ochratoxin have a harmful effect on early embryo development which results in decreased blastocyst proportion and delayed blastulation that impair subsequent embryonic development (Hsuuw et al., 2013; Somoskoi et al., 2016). However, no research has been done to explore the mechanism of ZEA toxicity to porcine early embryos and to propose a method of detoxification. Our study first examined the effects of different concentrations of ZEA on the development of 2-cell and blastocysts. The results showed that the damage to the embryos increased with the ZEA concentration. In addition, previous studies demonstrated that exposure to ZEA affected oocyte meiotic maturation in pig (Sambuu et al., 2011; Han et al., 2015), suggesting that the toxicity of ZEA to oocyte and embryo development is persistent. To find an approach to reduce the toxicity of ZEA on embryos we added melatonin. Since melatonin is shown to improve oocyte maturation rate, fertilization rate, embryos quality and pregnancy outcome without significant physiological side effects in animal experiments and human clinical studies (Paterson and Foldes, 1994; Fernando and Rombauts, 2014). Previous studies have shown that the most effective concentration of melatonin for oocyte maturation and embryonic development is 0.001 μM ∼ 0.1 μM, and we selected 0.1 μM to prevent ZEA-induced damage (Tian et al., 2014). Our results showed that melatonin had a protective effect on the embryonic development abnormalities caused by ZEA exposure in pigs.

In order to find out the possible mechanism of abnormal embryonic development caused by ZEA, we first examined the mitochondria, and the result showed that ZEA caused mitochondrial dysfunction, while melatonin effectively prevented ZEA-induced mitochondrial dysfunction. Mitochondria are essential for oocyte maturation, fertilization and embryonic development. Mitochondrial dysfunction leads to a decline in oocyte quality and affects embryonic development (Babayev and Seli, 2015). ZEA could cause mitochondrial damage in cultured swine small intestine IPEC-J2 cell (Fan et al., 2017). In addition, studies also have shown that feeding mice with mycotoxins, such as DON, ZEA, and aflatoxin (AF), leads to abnormal mitochondrial distribution of oocytes, resulting in a decline in oocyte quality (Hou et al., 2014). Our results indicate that ZEA leads to abnormal embryonic development by causing mitochondrial dysfunction, and that melatonin supplementation can save mitochondrial damage.

Low levels of ROS are beneficial, facilitating adaptation to stress via signaling, whereas high levels of ROS are deleterious because they trigger oxidative stress (Scialo et al., 2017). And excessive ROS induced by environmental stress destroys the structure of cells and results in the cell death (Bayir and Kagan, 2008). While mitochondrial dysfunction can lead to oxidative stress that product excessive ROS (Zorov et al., 2014). In mouse oocytes, other Environmental Endocrine Disruptors like Bisphenol AF exposure caused increased levels of ROS resulting in oxidative stress (Ding et al., 2017). Therefore we next examined whether ZEA-exposed produced oxidative stress. Consistently, our data showed that ZEA-exposed lead to significant oxidative stress, while melatonin supplementation prevented the production of ROS. Therefore, our results indicate that ZEA causes mitochondrial dysfunction-induced oxidative stress. Oxidative stress can cause DNA damage, and DNA damage can induce multiple cell apoptosis (Chatterjee and Walker, 2017). Studies have shown that ZEA and its metabolites enhance ROS production and DNA damage in human hepatoma cells (HepG2 cells) in a dose-dependent manner (Tatay et al., 2017). And bisphenol AF could cause DNA damage in mouse oocytes and affect the maturation of oocytes (Ding et al., 2017). In our results, DNA damage occurred in porcine 2-cell stage after ZEA treated, while melatonin inhibited this ZEA-induced DNA damage.

Oxidative stress also causes autophagy and autophagy is a naturally regulated cellular mechanism that degrades unnecessary proteins and dysfunctional organelles (Lee et al., 2012; Ryter and Choi, 2013). When oxidative stress causes damage, chaperon-mediated autophagy is activated and oxidized proteins are removed to protect cells (Kaushik and Cuervo, 2006). However, when autophagy does not protect against oxidative stress damage, the cells will necrosis, apoptosis or autophagy leading to death (Chen et al., 2008, 2009). ZEA could upregulate the expression of LC3-II and Beclin-1 in on rat Leydig cells and induce a higher level of autophagy (Wang et al., 2014). Studies have shown that DON exposure induces excessive autophagy and delays meiosis in porcine oocytes (Han et al., 2016). In our results ZEA exposure induced excessive autophagy, and melatonin supplementation significantly reduced autophagy levels. Our results suggest that melatonin protects early embryos from oxidative stress induced autophagy.

## Conclusion

In conclusion, our results indicated that ZEA had toxic effects on parthenogenetic activated early porcine embryos, showing with mitochondrial dysfunction, DNA damage and oxidative stress, while melatonin could prevent embryonic damage caused by ZEA.

## Author Contributions

YX and K-HZ performed the experiments. M-HS, ML, XW, and YZ contributed to materials and agents. YX drafted the manuscript. YX and S-CS designed the experiments. All authors approved the manuscript submission.

## Conflict of Interest Statement

The authors declare that the research was conducted in the absence of any commercial or financial relationships that could be construed as a potential conflict of interest.
